# Immunological Profiling of CD8^+^ and CD8^−^ NK Cell Subpopulations and Immune Checkpoint Alterations in Early-Onset Preeclampsia and Healthy Pregnancy

**DOI:** 10.3390/ijms25158378

**Published:** 2024-07-31

**Authors:** Laszlo Szereday, David U. Nagy, Fanni Vastag, Livia Mezosi, Matyas Meggyes

**Affiliations:** 1 Department of Medical Microbiology and Immunology, Medical School, University of Pécs, 12 Szigeti Street, 7624 Pécs, Hungary; szereday.laszlo@pte.hu (L.S.); livimezosi@gmail.com (L.M.); 2 Janos Szentagothai Research Centre, 20 Ifjusag Street, 7624 Pécs, Hungary; 3 Institute of Geobotany/Plant Ecology, Martin-Luther-University, Große Steinstraße 79/80, D-06108 Halle (Saale), Germany; davenagy9@gmail.com; 4 Department of Obstetrics and Gynaecology, Medical School, University of Pécs, 7624 Pécs, Hungary; vfanny92@gmail.com

**Keywords:** early-onset preeclampsia, NK cells, CD8, immune checkpoint pathways, TIGIT, CD226, LAG-3

## Abstract

Despite the numerous studies on the clinical aspects of early-onset preeclampsia, our understanding of the immunological consequences of inadequate placenta development remains incomplete. The Th1-predominance characteristic of early-onset preeclampsia significantly impacts maternal immunotolerance, and the role of immune checkpoint molecules in these mechanisms is yet to be fully elucidated. Our study aims to fill these crucial knowledge gaps. A total of 34 pregnant women diagnosed with early-onset preeclampsia and 34 healthy pregnant women were enrolled in this study. A mononuclear cell fragment from the venous blood was separated and frozen. The CD8^+^ and CD8^−^ NK cell subpopulations were identified and compared to their immune checkpoint molecule expressions using multicolor flow cytometry. The serum CD226 levels were measured by ELISA. Based on our measures, the frequency of the CD8^−^ subpopulation was significantly higher than that of the CD8^+^ counterpart in both the NKdim and NKbright subsets. Significantly lower CD226 surface expressions were detected in the preeclamptic group compared to healthy women in all the investigated subpopulations. However, while no difference was observed in the level of the soluble CD226 molecule between the two groups, the CD112 and CD155 surface expressions were significantly different. Our study’s findings underscore the significant role of the CD8^+^ and CD8^−^ NK subpopulations in the Th1-dominated immune environment. This deepens our understanding of early-onset preeclampsia and suggests that each subpopulation could contribute to the compensation mechanisms and the restoration of the immunological balance in this condition, a crucial step toward developing effective interventions.

## 1. Introduction

Preeclampsia, also known as pregnancy toxemia, is a severe, human, pregnancy-specific disease that is most likely associated with implantation and affects 3–5% of pregnancies [1]. Its classic symptoms usually manifest after the 20th gestational week, including high blood pressure, proteinuria, and the development of edema [2]. The disease is caused by the improper development of the placenta and the vessels in the placenta, eventually leading to symptoms [3]. Early detection of the disease (before the onset of symptoms) can be crucial for the timely initiation of appropriate prenatal care, which can reduce the risk factors associated with preterm birth. However, no such method is currently known. The current understanding suggests that early-onset (EO) preeclampsia, diagnosed before 34 weeks of pregnancy, might be linked to problems with implantation and abnormal placental development even before symptoms appear [4]. As the fetus grows rapidly after 20 weeks, the malfunctioning placenta cannot compensate for it, leading to the characteristic symptoms of preeclampsia (high blood pressure, proteinuria) [5]. Currently, the only therapy for the EO form of the disease is the artificial delivery of the fetus, so they are all born prematurely.

Once primarily classified as belonging to only the innate immune system, natural killer (NK) cells are recognized for their unique ability to bridge the gap between innate and adaptive immunity, allowing them to play a crucial role in combating malignant cells and viruses [6,7]. NK cells have unique skills to identify and eliminate abnormal cells, like those infected with viruses or transformed cells, without prior sensitization. The complex communication between NK cells and their microenvironment is essential for maintaining the body’s homeostasis and plays important roles in pregnancy and immune regulation. Peripheral blood NK cells comprise 5–10% of the peripheral lymphocytes. The major subset (over 90%) of the peripheral NK cells, called NKdim cells, which express a low level of the CD56 surface molecule, are primarily responsible for killing infected or abnormal cells [8]. The other approximately 10% of the NK cells form the NKbright subset, which has the surface CD56 molecule in high levels and plays a different role, mainly regulating the immune system [9]. The presence of the CD8 molecule on the surface of NK cells hints at a possible connection or intermediate stage between NK cells and cytotoxic T cells. These CD8^+^ NK cells have been observed in various immunological situations, including viral infections, autoimmune disorders, and cancer [10,11,12]. The importance of the CD8 molecule on the surface of NK cells is not fully understood. However, several investigations examined it to better understand its functions and contributions to immune responses.

Immune checkpoint (IC) molecules are essential in immunological regulation and maintain the balance between pro- and anti-inflammatory immune responses. Following a receptor–ligand interaction, a co-stimulatory or co-inhibitory signal is mediated, influencing the receptor-expressing cell’s function toward inflammation or tolerance. However, scientific data about the IC molecules are considerable, especially the IC inhibitors [13,14] in tumor immunology. Still, much less information is available regarding reproductive immunology, and these findings are often controversial [15]. T cell immunoglobulin and ITIM domain (TIGIT) is an inhibitory IC receptor expressed on activated T cells, including CD4^+^ T cells, regulatory T cells, natural killer T cells (NKT cells), and NK cells [16,17,18]. Numerous data suggest that TIGIT could inhibit the effector function of CD8^+^ T cells, down-regulate the cytotoxicity of NK cells [19,20], and decrease the T cell receptor expression [21]. The co-stimulatory counterpart of TIGIT is the CD226 (DNAM-1) surface receptor. TIGIT and CD226 compete to bind to CD155 (poliovirus receptor) and CD112 (PVRL2) [22]. The activator CD226 is found mainly on the surface of NK cells, CD8^+^ T cells, CD4^+^ T cells, and monocytes and plays a crucial role in the induction of proper NK and CD8^+^ T cell-mediated immune responses [23]. Blocking the CD226 receptor on NK cells can reduce their function, including cytokine production and activity, which inhibits the elimination of tumor cells [24,25]. The interaction of CD155 with CD226 mediates the activatory signals, while CD155/TIGIT connection transmits inhibitory signals to the receptor-expressed cells [20].

A recent study by our group examined the TIGIT, CD226, CD112, and CD155 immune checkpoint molecules in the peripheral blood mononuclear cells of women diagnosed with early-onset preeclampsia [26,27,28]. We found significantly decreased CD226 expression by the T-cell, NK cell, and NKT cell populations, together with an increased CD112 and CD155 expression by monocytes, in preeclamptic women compared to healthy controls [26,27]. This encouraged us to investigate the NK cell subpopulations further based on CD8 positivity to understand their role in early-onset preeclampsia.

LAG-3 is also an inhibitory surface receptor on activated CD4^+^ T, CD8^+^ T, and Treg cells. Two potential ligands have been identified for LAG-3, which are present in the tumor microenvironment [29]: liver sinusoidal endothelial cell lectin (LSECtin) and galectin-3 (Gal-3). Interaction between LAG-3 and LSECtin can inhibit IFN-γ release by effector T cells and increase IL-10 production by Tregs in melanoma [30]. A growing body of evidence proposed that LAG-3 signaling directly inhibits the primary activation of T cells, and the blockade of LAG-3 in T cells induces increased proliferation and cytokine production. The connection of Gal-3 and LAG-3 is an important mechanism in suppressing tumor-specific CD8^+^ T cells [31].

According to these data, the TIGIT/CD226/CD112/CD155 and LAG-3/Gal-3 IC pathways are essential for maintaining immunological tolerance. Furthermore, they could prevent the immune system from attacking healthy tissues. However, their precise role in reproduction, particularly in EO preeclampsia, remains largely unexplored.

## 2. Results

### 2.1. Identification and the Frequency of CD8^+^ and CD8^−^ NK Subpopulations in Healthy Pregnancy and EO Preeclampsia

**Figure 1:**
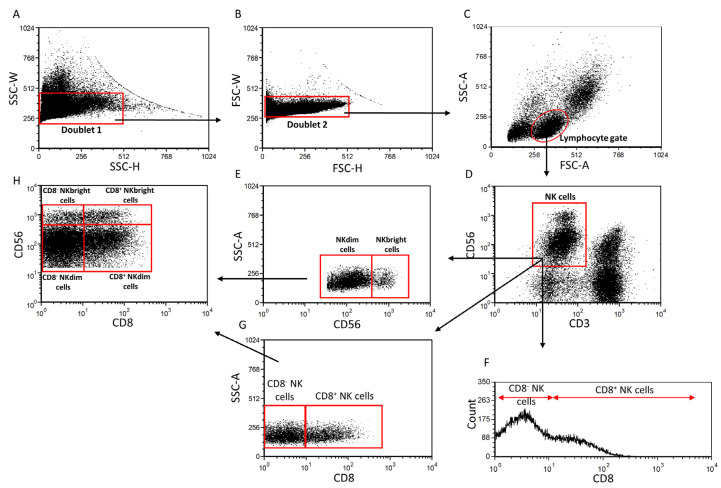
Flow cytometric gating strategy. Following a doublet exclusion (**A**,**B**), the lymphocyte gate was created using FSC-A/SSC-A parameters (**C**). The NK cell population was gated from the lymphocyte gate based on the CD3^−^/CD56^+^ combination (**D**). From the NK cells based on the density of the CD56 receptor, the NKdim and NKbright subpopulations were separated (**E**). From the NK cell subsets based on the presence of the CD8 receptor (**F**,**G**), further subpopulations were differentiated (**H**).

Multicolor flow cytometric analyses were used to characterize the investigated NK cell subsets, and the following gating strategy was applied. After a doublet exclusion (Figure 1A,B), the lymphocyte gate was determined based on the FSC-A/SSC-A parameters (Figure 1C). Different NKdim and NKbright cell subpopulations from the lymphocyte gate were separated from the NK cell population based on the density of the surface CD56 molecule (Figure 1D,E). Based on the presence of the CD8 surface receptor, further NK cell subsets were differentiated from the NK subpopulations (Figure 1F–H).

**Figure 2:**
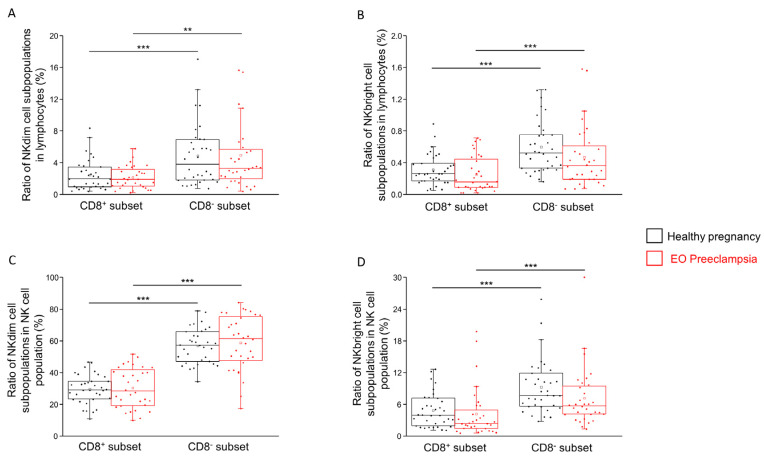
The frequency of different NKT subpopulations in EO preeclampsia and healthy pregnancy. The frequency of the CD8^+^ and CD8^−^ NKdim and NKbright subpopulations in the lymphocyte gate (**A**,**B**) and the CD3-CD56^+^ NK cell gate (**C**,**D**) in EO preeclamptic patients and healthy pregnant women. The solid bars represent the medians of 34 and 32 determinations, respectively. The boxes indicate the interquartile ranges, while the whiskers represent the variability of the minimum, maximum and any outlier data points compared to the interquartile range. The middle square within the box represents the mean value. Significant differences with *p*-values < 0.01 *** and < 0.03 ** are indicated.

Analyzing the frequency of the examined NK cell subpopulations, it can be generally stated that the frequencies of the CD8^−^ NKdim and CD8^−^ NKbright subsets are significantly higher compared to the CD8^+^ counterparts both in healthy pregnancy and in EO preeclampsia (Figure 2A–D). Further statistical difference between the investigated groups was not detected.

### 2.2. The Expression Profile of TIGIT and CD226 IC Receptors by CD8^+^ and CD8^−^ NK Subpopulations in Healthy Pregnancy and EO Preeclampsia

**Figure 3:**
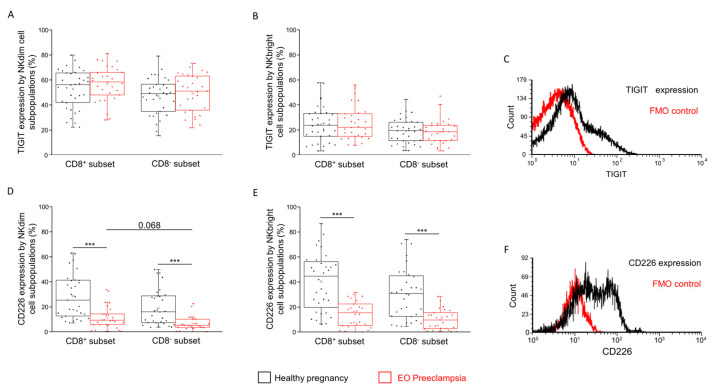
TIGIT and CD226 expression by CD8^+^ and CD8^−^ NK cell subpopulations in EO preeclampsia and healthy pregnancy. TIGIT receptor expression (**A**,**B**) and CD226 receptor expression (**D**,**E**) by the CD8^+^ and CD8^−^ NKdim and NKbright cell subpopulations in EO preeclamptic patients and healthy pregnant women. The solid bars represent the medians of 34/29 (in TIGIT) and 31/27 (in CD226) determinations, respectively. The boxes indicate the interquartile ranges, while the whiskers represent the variability of the minimum, maximum and any outlier data points compared to the interquartile range. The middle square within the box represents the mean value. Significant differences with *p*-values < 0.01 *** are indicated. Representative FACS histograms show the TIGIT surface marker (**C**) and CD226 surface molecule (**F**) expression by cells in the lymphocyte gate. Fluorescent minus one (FMO) control was used to determine the TIGIT and CD226 expression.

Comparing the inhibitory IC receptor expression, no statistical difference was detected in the surface level of TIGIT among the investigated subpopulations (Figure 3A,B).

In contrast, the activatory CD226 IC receptor expression was significantly lower in the EO preeclamptic groups in all the investigated subpopulations compared to the healthy group (Figure 3D,E). Another novel observation is a decreased CD8^−^ NKdim CD226 expression tendency compared to the CD8^−^ counterpart in the EO preeclampsia group (Figure 3D).

**Figure 4:**
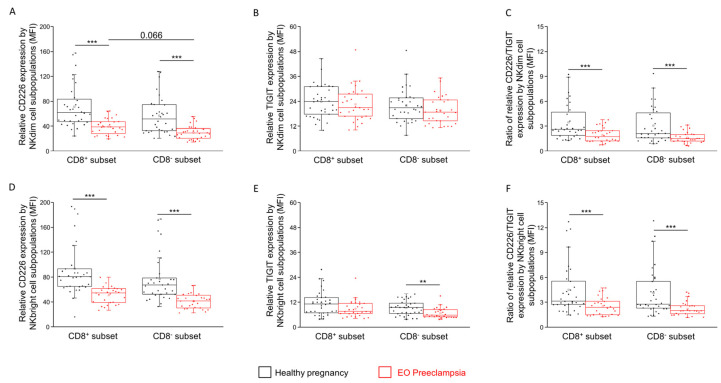
Relative TIGIT and CD226 expression CD8^+^ and CD8^−^ NK cell subpopulations in EO preeclampsia and healthy pregnancy. Mean fluorescent intensity (MFI) of the CD226 (**A**) and the TIGIT (**B**) receptors by the NKdim and CD226 (**D**) and the TIGIT (**E**) receptors by the NKbright subpopulations. in EO preeclamptic patients and healthy pregnant women. The ratio of the CD226/TIGIT mean fluorescent intensity value on the CD8^+^ and CD8^−^ NKdim (**C**) and NKbright (**F**) subpopulations. The solid bars represent the medians of 32/28 (in CD226) and 34/29 (in TIGIT) determinations, respectively. The boxes indicate the interquartile ranges, while the whiskers represent the variability of the minimum, maximum and any outlier data points compared to the interquartile range. The middle square within the box represents the mean value. Significant differences with *** and ** *p*-values < 0.03 are indicated.

Analyzing the relative expression of the mean fluorescent intensity (MFI), the above-mentioned IC receptors’ significantly lower CD226 MFI was measured in EO preeclamptic patients in all the NK subpopulations compared to the healthy donors (Figure 4A,D). In the case of the NKdim subpopulations, the CD226 MFI shows a decreased tendency in the CD8^−^ subset compared to the CD8^+^ subset (Figure 4A). Comparing the TIGIT MFI, no statistical difference was detected among the investigated groups (Figure 4B,E). The CD226/TIGIT MFI ratio on the surface of the NK cell subpopulations showed a significant decrease in all the investigated NK subpopulations in EO preeclampsia compared to the healthy controls (Figure 4C,F).

### 2.3. The Surface Expression of CD112 and CD155 IC Ligands by CD8^+^ and CD8^−^ NK Subpopulations in Healthy Pregnancy and EO Preeclampsia

**Figure 5:**
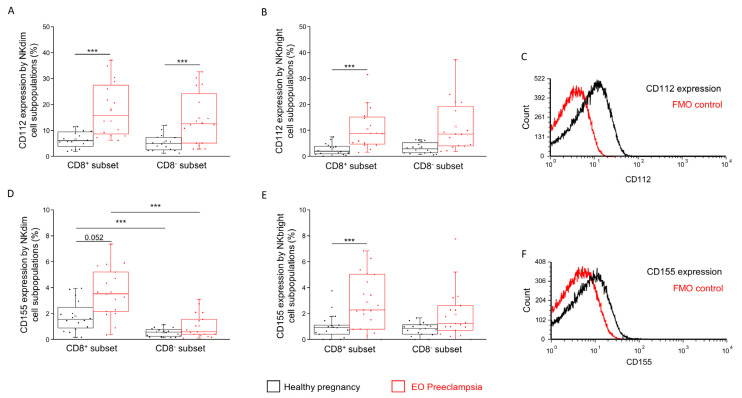
CD112 and CD155 expression by CD8^+^ and CD8^−^ NK cell subpopulations in EO preeclampsia and healthy pregnancy. CD112 ligand expression (**A**,**B**) and CD155 ligand expression (**D**,**E**) by the CD8^+^ and CD8^−^ NKdim and NKbright cell subpopulations in EO preeclamptic patients and healthy pregnant women. The solid bars represent the medians of 16/16 (in CD112) and 17/19 (in CD155) determinations, respectively. The boxes indicate the interquartile ranges, while the whiskers represent the variability of the minimum, maximum and any outlier data points compared to the interquartile range. The middle square within the box represents the mean value. Significant differences with *p*-values < 0.01 *** are indicated. Representative FACS histograms show the CD112 surface marker (**C**) and CD155 surface molecule (**F**) expression by cells in the lymphocyte gate. Fluorescent minus one (FMO) control was used to determine the CD112 and CD155 expression.

Analyzing the surface presence of CD112, significantly higher expression values were measured in both EO preeclamptic NKdim subpopulations compared to the values from the healthy pregnant group (Figure 5A). In the case of the NKbright subpopulation, the CD112 surface expression was elevated in only the EO preeclamptic CD8^+^ subset compared to the healthy CD8^+^ NKbright subset (Figure 5B).

Examining the other ligand in the case of NKdim cells, the surface expression of CD155 was significantly lower in the CD8^−^ subsets compared to the CD8^+^ counterparts both in EO preeclampsia and in healthy pregnancy (Figure 5D). A further significant observation is the higher CD155 expression by the EO preeclamptic CD8^+^ NKdim subset compared to the CD8^+^ counterpart in a healthy pregnancy (Figure 5D). In the case of the NKbright cells, significantly increased CD155 expression was measured by the CD8^+^ NKdim subpopulation in EO preeclampsia compared to the CD8^+^ NKdim cells from healthy conditions (Figure 5E).

### 2.4. The Expression of LAG-3 and Galectin-3 by CD8^+^ and CD8^−^ NK Subpopulations in Healthy Pregnancy and EO Preeclampsia

**Figure 6:**
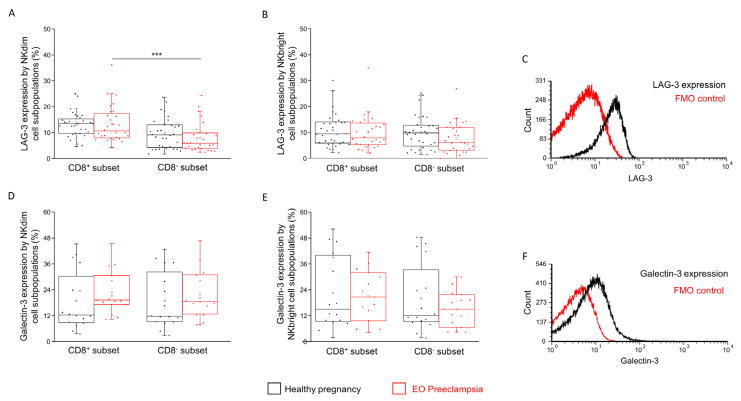
Surface expression of LAG-3 and intracellular expression of the Gal-3 molecule by CD8^+^ and CD8^−^ NK cell subpopulations in EO preeclampsia and healthy pregnancy. LAG-3 receptor expression (**A**,**B**) and Gal-3 ligand expression (**D**,**E**) by the CD8^+^ and CD8^−^ NKdim and NKbright cell subpopulations in EO preeclamptic patients and healthy pregnant women. The solid bars represent the medians of 32/28 (in LAG-3) and 16/15 (in Gal-3) determinations, respectively. The boxes indicate the interquartile ranges, while the whiskers represent the variability of the minimum, maximum and any outlier data points compared to the interquartile range. The middle square within the box represents the mean value. Significant differences with *p*-value < 0.01 *** are indicated. Representative FACS histograms show the LAG-3 surface marker (**C**) and Gal-3 intracellular molecule (**F**) expression by cells in the lymphocyte gate. Fluorescent minus one (FMO) control was used to determine the LAG-3 and Gal-3 expression.

Investigating part of another novel IC pathway, the LAG-3 surface receptor expression was significantly lower in the CD8^−^ NKdim subpopulations compared to the CD8^+^ counterpart, but only in EO preeclampsia (Figure 6A). No further difference was observed when comparing the NKbright subpopulations (Figure 6B).

The one possible ligand for the LAG-3 is the Gal-3 molecule. During our experiments, no statistical difference was detected in the Gal-3 expression by the investigated NK cell subpopulations (Figure 6D,E).

### 2.5. Intracellular Perforin and Granzyme B Content of the CD8^+^ and CD8^−^ NK Subpopulations in Healthy Pregnancy and in EO Preeclampsia

**Figure 7:**
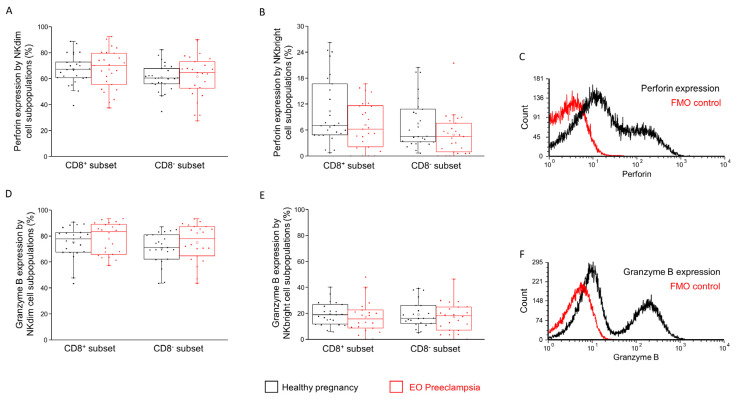
Intracellular perforin and granzyme B expression by CD8^+^ and CD8^−^ NK cell subpopulations in EO preeclampsia and healthy pregnancy. Perforin molecule content (**A**,**B**) and granzyme B molecule content (**D**,**E**) in the CD8^+^ and CD8^−^ NKdim and NKbright cell subpopulations in EO preeclamptic patients and healthy pregnant women. The solid bars represent the medians of 26/25 (in perforin) and 23/22 (in granzyme B) determinations, respectively. The boxes indicate the interquartile ranges, while the whiskers represent the variability of the minimum, maximum and any outlier data points compared to the interquartile range. The middle square within the box represents the mean value. Representative FACS histograms show the cells’ perforin expression (**C**) and granzyme B expression (**F**) in the lymphocyte gate. Fluorescent minus one (FMO) control was used to determine the perforin and granzyme B expression.

The intracellular perforin and granzyme B expressions were compared in EO preeclamptic and healthy conditions to reveal the NK subpopulations’ cytotoxic potential. However, no statistical difference was observed in all the comparisons (Figure 7).

### 2.6. The Relationship between the Soluble Level of CD226 and the Relative Expression of CD226 by NK Cell Subpopulations in Healthy Pregnancy and in EO Preeclampsia

**Figure 8:**
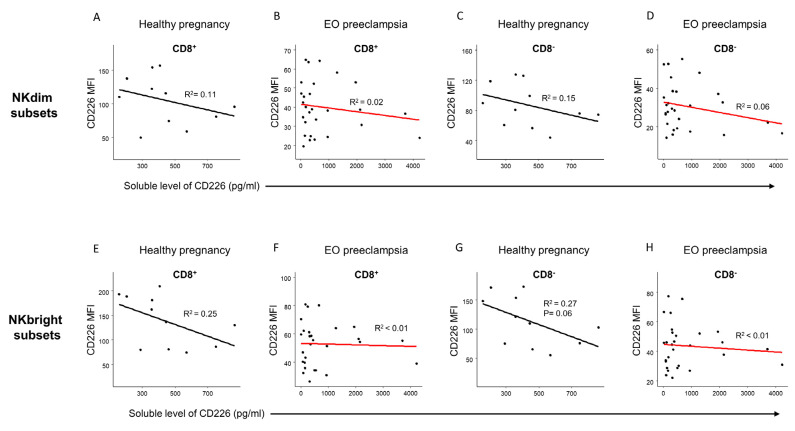
Regression analyses between the relative expression of CD226 by NK cell subpopulations and the soluble level of CD226 in EO preeclampsia and healthy pregnancy. Linear regression analyses between the soluble level of the CD226 molecule and the relative CD226 expression (MFI) by CD8^+^ and CD8^−^ NKdim (**A**–**D**) and NKbright (**E**–**H**) cell subpopulations in EO preeclamptic patients and healthy pregnant women. *p*-values and coefficients of determination (R2) were calculated in R.

No correlation or significant relation was detected between the relative CD226 expression by the different NK cell subsets and the serum CD226 (sCD226) levels (Figure 8).

## 3. Discussion

Healthy pregnancy involves a significant change in the extent of the maternal immune responses. In response to the presence of the fetus, the maternal immune system adapts to ensure its undisturbed development while maintaining its anti-microbial and anti-tumor functions. Maternal immune tolerance involves a delicate balance of various immune responses, and its disruption can lead to severe consequences for both the mother and the fetus. Immune checkpoint molecules are key molecules involved in immunoregulation. Through activatory and inhibitory functions, their task is to appropriately maintain immune responses. Their role has also been confirmed in autoimmune and tumor diseases, as well as in transplantation experiments. At the same time, their function in maternal immune tolerance mechanisms and EO preeclampsia is also very unclear. Therefore, our research focused on understanding the immune regulatory mechanisms in established EO preeclampsia.

Our findings indicate a significantly higher proportion of CD8^−^ NKdim and CD8^−^ NKbright subpopulations compared to the CD8^+^ counterparts in the peripheral blood of healthy and preeclamptic pregnant women. This may suggest that the CD8^−^ NK cells may represent a less immunologically active state. Ahmad et al. have shown that the CD8^+^ NK cell subpopulation exhibits more functional properties than the CD8^−^ counterpart, particularly in the context of HIV-1 infection [11]. This functional property difference likely extends to pregnancy and preeclampsia, where the immune system undergoes significant modulation. The persistence of maternal immune tolerance mechanisms, characteristic of the second trimester, might explain the observed proportion differences in the early third trimester when sampling occurred. This tolerance is critical for maintaining a healthy pregnancy, but in the context of EO preeclampsia, it could be disrupted, leading to an inflammatory immune environment.

No significant difference was observed in the proportions of the potentially more active CD8^+^ NK cell subpopulations between healthy and preeclamptic pregnancies. This suggests that while the proportion of CD8^−^ NK cells differs, their relative inactivity or altered functionality does not significantly vary, potentially contributing to the inflammatory environment characteristic of EO preeclampsia.

We, therefore, focused on the expression of activatory and inhibitory IC receptors. A comparison of the inhibitory TIGIT receptor expression revealed no significant differences between the NK subpopulations or the investigated groups. However, CD226, the activatory counterpart of the TIGIT, showed a significant decrease in surface and relative expression in the EO preeclamptic group in both the CD8^+^ and CD8^−^ subpopulations. This decrease may represent a compensatory mechanism to counteract the predominant Th1 immune responses in EO preeclampsia, adversely affecting maternal immunotolerance. By reducing the activating CD226 receptor, the body might aim to repress NK cell function to mitigate excessive inflammation [32]. The binding activity of TIGIT and CD226 to their ligands, CD112 and CD155, further supports this hypothesis [20]. Increased expression of CD112 and CD155 on NKdim and CD8^+^ NKbright cells in EO preeclampsia suggests a shift toward an inhibitory signal via the TIGIT receptor. This interaction could help counterbalance the inflammatory immune environment by reducing NK cell activity, thus providing an additional layer of immune regulation during the pathological progression of EO preeclampsia.

In our additional analysis of NKT cells, we identified novel and significant differences among the ratios of CD8^+^, CD4^+^, double-positive, and double-negative NKT cell subpopulations, and in the expression patterns of PD-1, LAG-3, TIGIT, and CD226 receptors [27]. These differences were particularly pronounced in the expression of CD112, PD-1, LAG-3, and CD226 MFI values between the early-onset preeclamptic and healthy pregnant groups [27]. These results suggest that these different NKT subpopulations act differently from each other under the altered immune conditions characteristic of early-onset preeclampsia. However, the CD8^+^ subpopulation shares similarities with the NK cell counterparts [27]. Furthermore, reduced TIGIT and granzyme B expressions were only measured in preeclamptic CD8^+^ T cells compared to healthy pregnant women [26]. A decreased level of the activatory receptor CD226 in effector lymphocytes accompanied by an elevated surface presence of the CD112 and CD155 ligands in monocytes could promote the TIGIT/CD112 and/or TIGIT/CD155 ligation, which mediates inhibitory signals similarly to our current findings [26].

When examining another inhibitory IC pathway, we measured significantly higher LAG-3 receptor expression on the CD8^+^ NKdim cells than the CD8^−^ subpopulation, but only in the EO preeclamptic group. This may also be related to regulating the potentially more active CD8^+^ NKdim subpopulation in EO preeclamptic conditions.

As cytotoxic cells, NK cells produce intracellular granules that destroy infected or tumor cells. Very few publications have examined the expression of perforin and granB in CD8^+^ and CD8^−^ NK cells, and those that have done have been contradictory [11,12]. A recent publication suggests that the CD8^+^ NK subpopulation is potentially more cytotoxic, supported by the higher proportion of perforin- and granB+ CD8^+^ NK cells in HIV-infected and HIV-seronegative control groups [11]. At the same time, McKinney et al. did not find a difference when comparing the cytotoxic granules of these CD8^+^ and CD8^−^ NK subpopulations in sclerosis multiplex patients [12]. Following our intracellular flow cytometric measurements, we did not detect any significant difference in the content of the cytotoxic granules. Since the intracellular content of these molecules does not necessarily reflect their capacity for degranulation, the evaluation of the CD107a marker should be measured with perforin and granB, but a limited number of fluorescent channels were available in the flow cytometer we used.

A recent study reported that in cutaneous T-cell lymphoma, the reduced expression of CD226 by the CD8^+^ T and NK cell populations was in line with the higher level of sCD226 in the serum. With further examinations, they found a negative correlation between the serum level of CD226 and the ratio of CD226-expressing CD56+ cells among the whole CD56+ cell population, which suggests that the soluble CD226 molecule might have originated from the membrane of the CD56+ NK- and CD8^+^ T cells [33]. However, we did not detect a significant relationship between the serum level of sCD226 and the surface expression of CD226 by the investigated NK subsets. The circulated CD226 may interact with the higher level of CD112 and CD155 ligand molecules on the surface of the monocyte subpopulations in preeclamptic women [26]. It might contribute to the Th1 predominance in EO preeclampsia by blocking the interaction with surface TIGIT receptors by the effector cell subsets.

While our study offers valuable insights, mentioning some limitations is important. First, our focus on peripheral blood cells might have missed immune activation in the maternal–fetal interface and their unique environments. Second, the limited availability of blood samples restricted our range of experiments. Third, longitudinal examinations were not performed, as we could not track the immune cell responses over time. This could have provided valuable information about how the immune system changes.

## 4. Materials and Methods

### 4.1. Patients

Collaborating with the Department of Obstetrics and Gynaecology, University of Pecs, 30 pregnant women diagnosed with the classic symptoms of preeclampsia (hypertension, proteinuria) were recruited in this case-control study (Table 1). Following the ISSHP criteria, preeclampsia was defined as new-onset hypertension (systolic blood pressure ≥ 140 mmHg or diastolic blood pressure ≥90 mmHg on at least two occasions, 4 h apart, within 24 h) before 34 weeks of gestation, accompanied by significant proteinuria in women with previously normal blood pressure (≥30 mg/mol protein in 24 h urine collection in the absence of urinary tract infection).

A total of 40 healthy pregnant women with appropriately matched gestational age were recruited from the Department of Obstetrics and Gynaecology, University of Pecs, to form the control group (Table 1).

Meticulous health assessments were conducted for all the participating females. This evaluation confirmed they had no significant medical history, were not taking any medications (including hormonal birth control) and had not experienced recent illnesses. Additionally, women with complications from pregnancy, infections, pre-existing conditions, in vitro fertilization, immune disorders, diabetes, or AIDS were excluded. Finally, none of the participants was a tobacco smoker.

### 4.2. Sample Collection, PBMC Separation, and Cryopreservation

A total of 20 mL of venous blood was collected from every participant into heparinized tubes, and another 10 mL of venous blood was taken to native tubes to collect serum samples.

The peripheral blood mononuclear cells (PBMCs) were separated from the heparinized venous blood on a Ficoll-Paque density (GE Healthcare, Chicago, IL, USA) gradient. The samples were washed in RPMI 1640 medium (Lonza, Basel, Switzerland), and the number of cells was determined. Following another washing step, the PBMCs were resuspended in inactivated human serum containing 10% DMSO (Sigma-Aldrich, St. Louis, MA, USA) for cryoprotection. The cells were then aliquoted in cryovials and stored at −80 °C. Before fluorescent labeling, the cryovials were thawed rapidly at 37 °C and washed twice with RPMI 1640 medium to remove the DMSO solution.

### 4.3. Flow Cytometric Measurement

A total of 106 thawed PBMCs were labeled with a combination of fluorochrome-conjugated monoclonal antibodies (Table 2) for 30 min at room temperature (RT) in complete darkness. Next, the cells were washed in phosphate-buffered saline (PBS) (BioSera, Cholet, France) and resuspended in 300 µL PBS containing 1% paraformaldehyde (PFA) and stored at 4 °C in darkness until flow cytometric analysis. The flow cytometric measurements were performed using a BD FACS Canto II flow cytometer (BD Immunocytometry Systems, Erembodegem, Belgium) equipped with the BD FACS Diva V6. Software (BD Biosciences, Franklin Lakes, NJ, USA) for data acquisition. FCS Express V4 (De Novo Software, Pasadena, CA, USA) was used for the data analyses.

### 4.4. Intracellular Staining

After the surface staining, the samples were washed with PBS and fixed in 4% PFA for 10 min at RT in complete darkness. Next, the cells were washed again with PBS and permeabilized using a 1:10 dilution of FACS Permeabilizing Solution 2 (BD Biosciences) for 10 min at RT in darkness. Following the washing step, the samples were incubated with FITC-conjugated anti-human granzyme B and PE-Cy7-conjugated anti-human perforin antibodies for 30 min at RT in darkness. The cells were washed with PBS, fixed with 1% PFA and stored at 4 °C in darkness until further FACS analysis.

### 4.5. ELISA

Using sandwich enzyme-linked immunosorbent assays (ELISAs, R&D Systems, Bio-Techne, DY666-05, Ancillary Reagent kit 2: R&D Systems, Bio-Techne, DY008, Specificity: no cross-reactivity or interference with 50 ng/mL recombinant human CD155/Fc Chimera CD96/Fc Chimera Integrin β2 Nectin-2/Fc Chimera PVRIG/Fc Chimera and recombinant mouse DNAM-1/Fc Chimera; Sensitivity: 62.5–4000 pg/mL), the CD226 concentrations were measured following previously published methods [34]. A 96-well flat-bottom microplate was coated overnight with 100 μL/well anti-human CD226 antibody at RT. Subsequently, the plate was washed with 3 × 400 μL/well of wash buffer and blocked with 400 μL/well of reagent diluent for an hour at RT. After another three-washing step, 100 μL of serially diluted standards and thawed serum samples were added to the plate and incubated for two hours at RT. Following incubation, the wells were washed again, and 100 μL of detection antibody was added to the wells for two hours at RT. After another three-washing step, 100 μL/well of streptavidin HRP was pipetted for 20 min. Following the final three washes, the plate was developed for 20 min with a 1:1 mixture of substrate reagents A (H_2_O_2_) + B (tetramethylbenzidine) in darkness. The reaction was then terminated with a Stop solution. The absorbance of the test plates was read immediately at 450 nm or 450 nm with a reference filter of 540 nm using a BMG SPECTOstar Nano spectrophotometer (BMG Labtech, Ortenberg, Germany). Following background subtraction, standard curves were generated using 4-parametric logistic analysis, and the CD226 concentrations were calculated using MARS Data Analysis Software version 3.32 (BMG Labtech, Ortenberg, Germany).

### 4.6. Statistical Analysis

To analyze the differences between the investigated parameters of the NKdim and NKbright subpopulations, linear models were run in R. Decisions on the transformation of the response variables depended on a visual inspection of the “model-checking plots” for the models with transformed vs. untransformed variables. Based on these plots, the normality of the residuals and the assumption of the homogeneity of variance were checked [35]. The variables were log-transformed. The explanatory variables were the two-way interaction effects of the cell types (CD8^+^ or CD8^−^) × investigated groups (healthy pregnancy and EO preeclampsia). A two-way ANOVA was used to test for statistical significance. Tukey’s post hoc tests were conducted to compare each cell type/status combination for the pair-wise comparisons.

Additionally, linear regression models were separately conducted for the ELISA and different CD8 measures across the groups of women. Each variable was log-transformed.

## 5. Conclusions

In conclusion, our study highlights the significant involvement of CD8^+^ and CD8^−^ NK cell subpopulations in the immunological environment of early-onset preeclampsia. We observed distinct alterations in the immune checkpoint molecule expressions between preeclamptic and healthy pregnancies, particularly the decreased surface expression of CD226 in the preeclamptic group. These findings suggest that the immune regulatory mechanisms, including the TIGIT/CD226 and LAG-3 pathways, play a crucial role in the immunotolerance processes disrupted in preeclampsia. Understanding these immune alterations provides a deeper insight into the pathogenesis of early-onset preeclampsia and may guide the development of targeted therapeutic strategies to restore immunological balance in affected pregnancies.

## Figures and Tables

**Table 1:** Demographic and gynecological data of the participating women.

	Healthy Pregnant Women	Early-Onset Preeclamptic Patients
**No. of patients**	40	30
**Age (years)**	32.50 (23–44)	30.37 (18–43)
**Gestational age at birth (weeks)**	39.12 ± 0.89	30.73 ± 2.55 *
**Gestational age at sampling (weeks)**	31.39 ± 3.67	30.17 ± 2.56
**Birth weight (gram)**	3453.03 ± 353.91	1328.62 ± 408.45 *

The results are expressed as the mean value ± standard deviation (SD) of the mean. Statistical analyses were performed using the independent sample *t*-tests. * *p* < 0.01 vs healthy pregnant women.

**Table 2:** Fluorochrome-conjugated monoclonal antibodies used in the study.

Antigen	Format	Clone	Isotype	Company	CAT
**CD3**	BV510	UCHT1	Mouse BALB/c IgG1, κ	BD Biosciences, Franklin Lakes, NJ, USA	563109
**CD8**	APC-H7	SK1	Mouse BALB/c IgG1, κ	BD Biosciences, Franklin Lakes, NJ, USA	560179
**CD56**	APC	B159	Mouse IgG1, κ	BD Biosciences, Franklin Lakes, NJ, USA	555518
**CD112**	PE	R2.525	Mouse IgG1, κ	BD Biosciences, Franklin Lakes, NJ, USA	551057
**CD155**	APC	SKII.4	Mouse IgG1, κ	BioLegend, San Diego, CA, USA	337618
**CD226**	BV421	DX11	Mouse BALB/c IgG1, κ	BD Biosciences, Franklin Lakes, NJ, USA	742493
**Galectin-3**	PE	B2C10	Mouse BALB/c IgG1, κ	BD Biosciences, Franklin Lakes, NJ, USA	565676
**LAG-3**	PerCp Cy5.5	11C3C65	Mouse IgG1, κ	BioLegend, San Diego, CA, USA	369312
**Granzyme B**	FITC	GB11	Mouse BALB/c IgG1, κ	BD Biosciences, Franklin Lakes, NJ, USA	560211
**Perforin**	PE-Cy7	dG9	Mouse IgG2b, κ	BioLegend, San Diego, CA, USA	308126
**TIGIT**	PE	A1553G	Mouse IgG2a, κ	BioLegend, San Diego, CA, USA	372704

## Data Availability

The data used to support the findings of this study are available from the corresponding author upon request.

## References

[1] Brown M.A., Magee L.A., Kenny L.C., Karumanchi S.A., McCarthy F.P., Saito S., Hall D.R., Warren C.E., Adoyi G., Ishaku S. (2018). Hypertensive Disorders of Pregnancy: ISSHP Classification, Diagnosis, and Management Recommendations for International Practice. Hypertension.

[2] Poon L.C., Shennan A., Hyett J.A., Kapur A., Hadar E., Divakar H., McAuliffe F., da Silva Costa F., von Dadelszen P., McIntyre H.D. (2019). The International Federation of Gynecology and Obstetrics (FIGO) initiative on pre-eclampsia: A pragmatic guide for first-trimester screening and prevention. Int. J. Gynaecol. Obstet..

[3] Litang Z., Hong W., Weimin Z., Xiaohui T., Qian S. (2017). Serum NF-κBp65, TLR4 as biomarker for diagnosis of preeclampsia. Open Med..

[4] Raymond D., Peterson E. (2011). A critical review of early-onset and late-onset preeclampsia. Obstet. Gynecol. Surv..

[5] Powers R.W., Jeyabalan A., Clifton R.G., Van Dorsten P., Hauth J.C., Klebanoff M.A., Lindheimer M.D., Sibai B., Landon M., Miodovnik M. (2010). Soluble fms-Like Tyrosine Kinase 1 (sFlt1), endoglin and placental growth factor (plgf) in preeclampsia among high risk pregnancies. PLoS ONE.

[6] Sun J.C., Lanier L.L. (2009). Natural killer cells remember: An evolutionary bridge between innate and adaptive immunity?. Eur. J. Immunol..

[7] Artis D., Spits H. (2015). The biology of innate lymphoid cells. Nature.

[8] Moretta A., Marcenaro E., Parolini S., Ferlazzo G., Moretta L. (2008). NK cells at the interface between innate and adaptive immunity. Cell Death Differ..

[9] Yang F., Zheng Q., Jin L. (2019). Dynamic Function and Composition Changes of Immune Cells During Normal and Pathological Pregnancy at the Maternal-Fetal Interface. Front. Immunol..

[10] Lucar O., Reeves R.K., Jost S. (2019). A Natural Impact: NK Cells at the Intersection of Cancer and HIV Disease. Front. Immunol..

[11] Ahmad F., Hong H.S., Jäckel M., Jablonka A., Lu I.-N., Bhatnagar N., Eberhard J.M., Bollmann B.A., Ballmaier M., Zielinska-Skowronek M. (2014). High frequencies of polyfunctional CD8^+^NK cells in chronic HIV-1 infection are associated with slower disease progression. J. Virol..

[12] McKinney E.F., Cuthbertson I., Harris K.M., Smilek D.E., Connor C., Manferrari G., Carr E.J., Zamvil S.S., Smith K.G.C. (2021). A CD8^+^ NK cell transcriptomic signature associated with clinical outcome in relapsing remitting multiple sclerosis. Nat. Commun..

[13] Chen X., Wu W., Wei W., Zou L. (2022). Immune Checkpoint Inhibitors in Peripheral T-Cell Lymphoma. Front. Pharmacol..

[14] Mao X.-C., Yang C.-C., Yang Y.-F., Yan L.-J., Ding Z.-N., Liu H., Yan Y.-C., Dong Z.-R., Wang D.-X., Li T. (2022). Peripheral cytokine levels as novel predictors of survival in cancer patients treated with immune checkpoint inhibitors: A systematic review and meta-analysis. Front. Immunol..

[15] Miko E., Meggyes M., Doba K., Barakonyi A., Szereday L. (2019). Immune Checkpoint Molecules in Reproductive Immunology. Front. Immunol..

[16] Yu X., Harden K., Gonzalez L.C., Francesco M., Chiang E., Irving B., Tom I., Ivelja S., Refino C.J., Clark H. (2009). The surface protein TIGIT suppresses T cell activation by promoting the generation of mature immunoregulatory dendritic cells. Nat. Immunol..

[17] Boles K.S., Vermi W., Facchetti F., Fuchs A., Wilson T.J., Diacovo T.G., Cella M., Colonna M. (2009). A novel molecular interaction for the adhesion of follicular CD4 T cells to follicular DC. Eur. J. Immunol..

[18] Johnston R.J., Comps-Agrar L., Hackney J., Yu X., Huseni M., Yang Y., Park S., Javinal V., Chiu H., Irving B. (2014). The immunoreceptor TIGIT regulates antitumor and antiviral CD8(+)T cell effector function. Cancer Cell.

[19] Stanietsky N., Rovis T.L., Glasner A., Seidel E., Tsukerman P., Yamin R., Enk J., Jonjic S., Mandelboim O. (2013). Mouse TIGIT inhibits NK-cell cytotoxicity upon interaction with PVR. Eur. J. Immunol..

[20] He W., Zhang H., Han F., Chen X., Lin R., Wang W., Qiu H., Zhuang Z., Liao Q., Zhang W. (2017). CD155T/TIGIT Signaling Regulates CD8^+^ T-cell Metabolism and Promotes Tumor Progression in Human Gastric Cancer. Cancer Res..

[21] Pauken K.E., Wherry E.J. (2014). TIGIT and CD226: Tipping the balance between costimulatory and coinhibitory molecules to augment the cancer immunotherapy toolkit. Cancer Cell.

[22] Linsley P.S., Greene J.L., Brady W., Bajorath J., Ledbetter J.A., Peach R. (1994). Human B7-1 (CD80) and B7-2 (CD86) bind with similar avidities but distinct kinetics to CD28 and CTLA-4 receptors. Immunity.

[23] Nabekura T., Kanaya M., Shibuya A., Fu G., Gascoigne N.R., Lanier L.L. (2014). Costimulatory molecule DNAM-1 is essential for optimal differentiation of memory natural killer cells during mouse cytomegalovirus infection. Immunity.

[24] Shibuya A., Campbell D., Hannum C., Yssel H., Franz-Bacon K., McClanahan T., Kitamura T., Nicholl J., Sutherland G.R., Lanier L.L. (1996). DNAM-1, a novel adhesion molecule involved in the cytolytic function of T lymphocytes. Immunity.

[25] Cluxton C.D., Spillane C., O’Toole S.A., Sheils O., Gardiner C.M., O’Leary J.J. (2019). Suppression of Natural Killer cell NKG2D and CD226 anti-tumour cascades by platelet cloaked cancer cells: Implications for the metastatic cascade. PLoS ONE.

[26] Szereday L., Nagy D.U., Csiszar B., Kevey D., Feik T., Meggyes M. (2021). Examination of the TIGIT, CD226, CD112, and CD155 immune checkpoint molecules in peripheral blood mononuclear cells in women diagnosed with early-onset preeclampsia. Biomedicines.

[27] Meggyes M., Feik T., Nagy D.U., Polgar B., Szereday L. (2023). CD8 and CD4 Positive NKT Subpopulations and Immune-Checkpoint Pathways in Early-Onset Preeclampsia and Healthy Pregnancy. Int. J. Mol. Sci..

[28] Meggyes M., Nagy D.U., Szigeti B., Csiszar B., Sandor B., Tamas P., Szereday L. (2020). Investigation of Mucosal-Associated Invariant T (MAIT) Cells Expressing Immune Checkpoint Receptors (TIGIT and CD226) in Early-Onset Preeclampsia. Eur. J. Obstet. Gynecol. Reprod. Biol..

[29] Le Mercier I., Lines J.L., Noelle R.J. (2015). Beyond CTLA-4 and PD-1, the Generation Z of Negative Checkpoint Regulators. Front. Immunol..

[30] Xu F., Liu J., Liu D., Liu B., Wang M., Hu Z., Du X., Tang L., He F. (2014). LSECtin expressed on melanoma cells promotes tumor progression by inhibiting antitumor T-cell responses. Cancer Res..

[31] Kouo T., Huang L., Pucsek A.B., Cao M., Solt S., Armstrong T., Jaffee E. (2015). Galectin-3 Shapes Antitumor Immune Responses by Suppressing CD8^+^ T Cells via LAG-3 and Inhibiting Expansion of Plasmacytoid Dendritic Cells. Cancer Immunol. Res..

[32] Marin-Acevedo J.A., Kimbrough E.O., Lou Y. (2021). Next generation of immune checkpoint inhibitors and beyond. J. Hematol. Oncol..

[33] Takahashi N., Sugaya M., Suga H., Oka T., Kawaguchi M., Miyagaki T., Fujita H., Inozume T., Sato S. (2017). Increased Soluble CD226 in Sera of Patients with Cutaneous T-Cell Lymphoma Mediates Cytotoxic Activity against Tumor Cells via CD155. J. Investig. Dermatol..

[34] Meggyes M., Nagy D.U., Feik T., Boros A., Polgar B., Szereday L. (2022). Examination of the TIGIT-CD226-CD112-CD155 Immune Checkpoint Network during a Healthy Pregnancy. Int. J. Mol. Sci..

[35] Crawley M.J. (2014). Statistics: An Introduction Using R.

